# Dihydroartemisinin-regulated mRNAs and lncRNAs in chronic myeloid leukemia

**DOI:** 10.18632/oncotarget.23274

**Published:** 2017-12-15

**Authors:** Xiang Li, Yue Gao, Qiang Zhang, Nan Hu, Dong Han, Shangwei Ning, Zhuo Ao

**Affiliations:** ^1^ CAS Center for Excellence in Nanoscience, National Center for Nanoscience and Technology, Beijing 100190, China; ^2^ College of Bioinformatics Science and Technology, Harbin Medical University, Harbin 150081, China; ^3^ Department of Traditional Chinese Medicine, Chengde Medical University, Chengde 066000, China

**Keywords:** dihydroartemisinin, chronic myeloid leukemia, long non-coding RNA, lncRNA-mRNA network, steroid biosynthesis

## Abstract

Chronic myelocytic leukemia (CML) is characterized by increased and unregulated growth of predominantly myeloid cells in the bone marrow, and accumulation of these cells in blood. We investigated the effects of an anti-malarial drug, dihydroartemisinin (DHA), on K562 CML cells. We identified 34 mRNAs and eight lncRNAs dysregulated following DHA treatment in pure and hemin-induced K562 cells. Up- or downregulation of these potential DHA targets increased with increasing DHA concentration. We also constructed and analyzed a DHA-related mRNA-lncRNA regulation network in K562 cells, and found that four DHA-modulated mRNAs regulated by four lncRNAs participated in the steroid biosynthesis pathway. Some estrogen-related drugs, such as tamoxifen, shared common targets with DHA. We inferred that DHA exerted anti-cancer effects on K562 cells by influencing estrogen levels. Our findings indicate that DHA has potential not only as an anti-malarial drug, but also as an anti-CML chemotherapeutic.

## INTRODUCTION

Leukemia is characterized by the malignant proliferation of hematopoietic cells with disrupted differentiation and apoptotic programs. Leukemia usually originates in bone marrow, leading to high numbers of abnormal white blood cells [1]. Treatment for chronic myelocytic leukemia (CML) commonly involves a combination of chemotherapy, radiation therapy, targeted therapy, and bone marrow transplantation, in addition to supportive and palliative care as needed [2, 3]. CML patient outcomes have steadily improved in recent years. Still, while many studies have revealed key leukemia- and CML-specific genes and pathways, our understanding of underlying disease mechanisms, and the subsequent development of new therapeutics, have progressed slowly [4].

Long non-coding RNAs (lncRNAs) have gained widespread attention in recent years due to their important biological functions and potential clinical significance [5]. lncRNAs have been implicated in the development and progression of numerous human diseases, including cancer [6–8] Multiple studies have associated lncRNAs with CML. For example, lncRNA CCD26 controls leukemia cell growth by regulating KIT expression [9]. Thus, strategies that modulate lncRNAs to regulate cell division, apoptosis, invasion, and metastasis may prove effective against CML.

Artemisinin, isolated from the plant, *Artemisia annua*, and an artemisinin derivative, dihydroartemisinin (DHA), have been developed as novel antimalarial drugs [10]. DHA, a safe and effective anti-malarial, is more water-soluble and has stronger biological activity than artemisinin. DHA also exhibits activity against several human cancers, especially leukemia [11–16]. DHA inhibited vascular endothelial growth factor (VEGF) expression and induced apoptosis in CML K562 cells [17].

The present study identified lncRNAs and mRNAs dysregulated in K562 cells treated with DHA. We also constructed DHA-associated lncRNA-mRNA interaction networks to explore the mechanisms by which DHA exerts its anti-CML effects. We found eight lncRNAs downregulated by DHA, four of which regulated mRNAs involved in steroid biosynthesis. Our results reveal mechanisms underlying the effects of DHA on CML, and suggest that DHA may be an effective anti-CML drug.

## RESULTS

### Dysregulated mRNAs in DHA-treated K562 cells

**Figure 1:**
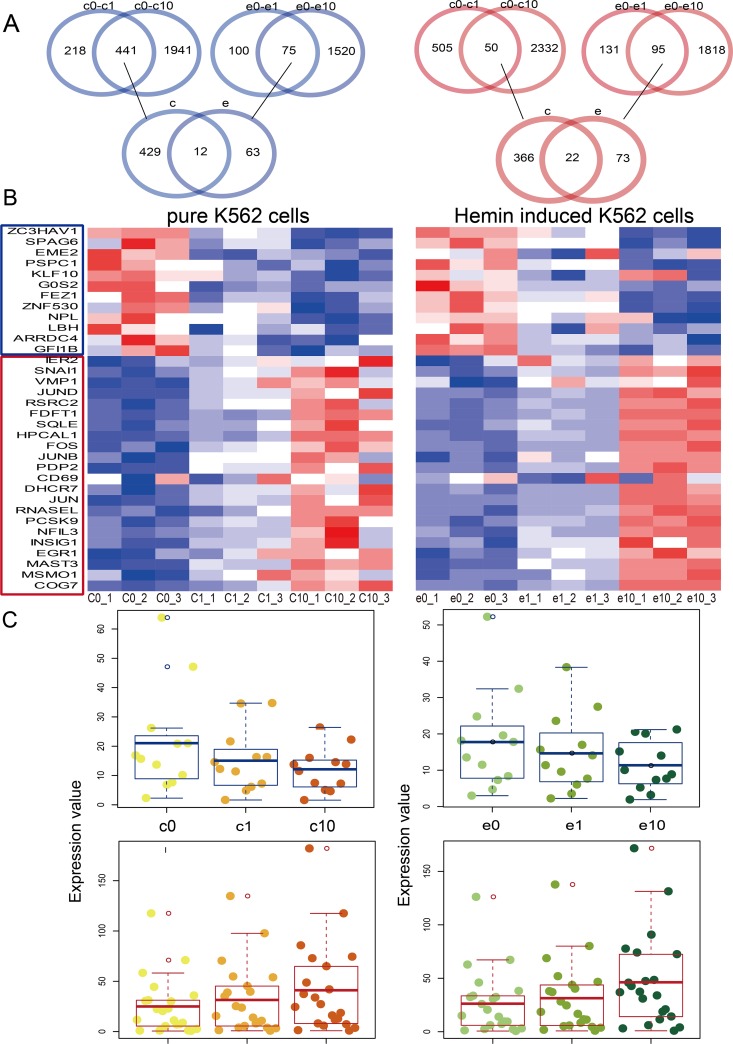
Dysregulated mRNAs in pure and hemin-induced K562 cells Venn diagrams showing the differentially expressed mRNAs shared among four groups, including pure K562 cells and those treated with 1 uM DHA (c0-c1), pure K562 cells and those treated with 10 uM DHA (c0-c10), hemin-induced K562 cells and those treated with 1 uM DHA (e0-e1), and hemin-induced K562 cells and those treated with 10uM DHA (e0-e10) **(A)** Heat maps of dysregulated mRNAs in pure and hemin-induced K562 cells **(B)** Red and blue colors in (A) and (B) represent upregulated and downregulated mRNAs, respectively. Boxplots showing differentially expressed mRNAs **(C)** Darker color represents treatment with higher DHA concentrations. Yellow and green represent pure and hemin-induced K562 cells, respectively.

We identified 34 differentially expressed mRNAs in pure and hemin-induced K562 cells treated with different DHA concentrations (1 or 10 μm) (Figure 1A). We classified the mRNAs, which were potential targets of DHA, as upregulated or downregulated based on fold change values (fold change >2 and <0.5, respectively). Twelve mRNAs were downregulated and 22 were upregulated (Figure 1A). Heat maps show mRNA expression in pure and hemin-induced K562 cells treated with different DHA concentrations (Figure 1B). We found that the level of mRNA up- or downregulation increased with increasing DHA concentration (Figure 1C). Previous studies have associated some of these DHA-regulated mRNAs with leukemia or CML (Supplementary Table 1). The zinc finger antiviral protein (ZAP, gene symbol ZC3HAV1) was originally identified as inhibiting the retrovirus, Moloney murine leukemia virus [18]. Sperm-associated antigen 6 (SPAG6) is a risk factor for childhood acute myelogenous leukemia [19].

### Dysregulated lncRNAs in DHA-treated K562 cells

**Figure 2:**
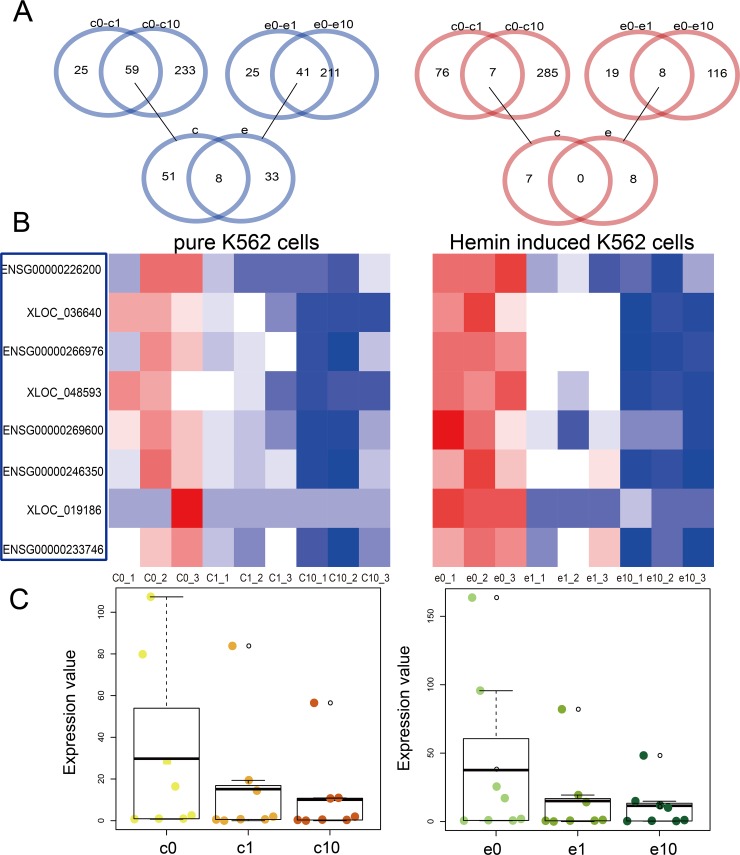
Dysregulated lncRNAs in pure and hemin-induced K562 cells Venn diagrams showing differentially expressed lncRNAs shared among four groups, including pure K562 cells and those treated with 1 uM DHA (c0-c1), pure K562 cells and those treated with 10 uM DHA (c0-c10), hemin-induced K562 cells and those treated with 1 uM DHA (e0-e1), and hemin-induced K562 cells and those treated with 10 uM DHA (e0-e10) **(A)** Heat maps of dysregulated lncRNAs in pure and hemin-induced K562 cells **(B)** Red and blue colors in (A) and (B) represent upregulated and downregulated lncRNAs, respectively. Boxplots showing differentially expressed lncRNAs **(C)** Darker color represents treatment with higher DHA concentrations. Yellow and green represent pure and hemin-induced K562 cells, respectively.

Using the same methods as for mRNA identification, we identified eight lncRNAs downregulated following DHA treatment (Figure 2A). Heat maps showed differential lncRNA expression in pure and hemin-induced K562 cells treated with different DHA concentrations (Figure 2B). As with our mRNA observations, lncRNA downregulation increased with increasing DHA concentration (Figure 2C). Some dysregulated lncRNAs identified in this study have been previously associated with leukemia. SGMS1-AS1, a lncRNA located on the antisense strand of sphingomyelin synthase 1 (SGMS1), may play a role in D609-induced apoptosis in U937 human monocytic leukemia cells [20].

### DHA-related mRNA-lncRNA regulation network analysis

**Figure 3:**
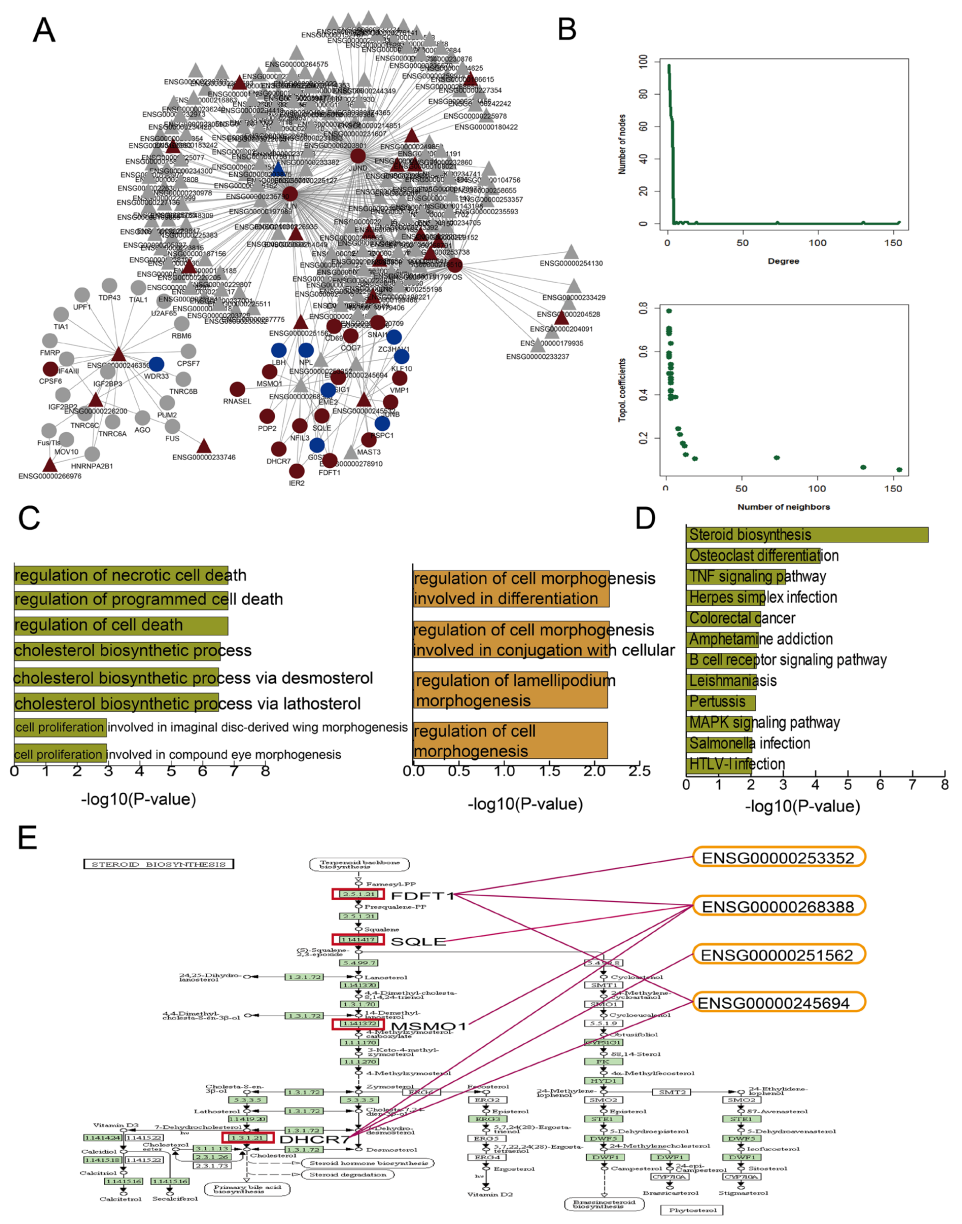
DHA-related mRNA-lncRNA network and functional analysis DHA related mRNA-lncRNA regulation network **(A)** Nodes represent candidate DHA targets in K562 cells. Circular and triangle nodes represent mRNAs and lncRNAs, respectively. Red and blue colors represent up- and downregulation, respectively. Basic network features, including degree and topological coefficients **(B)** Pathways enriched for differential mRNAs **(C)** and GO terms enriched for differential mRNAs (green) and lncRNAs (yellow) **(D)** are ranked by -log10(P) and shown as bar plots. The steroid biosynthesis pathway shows four key dysregulated mRNAs (red rectangles) regulated by four dysregulated lncRNAs **(E)**.

We integrated multiple data sources to construct an mRNA-lncRNA regulation network containing 238 nodes (191 lncRNAs and 47 mRNAs) and 428 edges (Figure 3A). The network exhibited the scale-free topology of a transcriptional regulatory network. The degrees, topological coefficients, and neighborhood connectivity followed a scale-free distribution and indicated that the network adheres to the small-world phenomenon (Figure 3B).

GO and pathway analyses using the DHA-regulated mRNAs and lncRNAs (Figure 3C) showed enrichment in GO terms associated with cell morphogenesis. Previous studies have associated leukemia development with cell morphogenesis [20, 21]. Other enriched GO terms suggested that CML cells, as in many other cancers, are protected from programmed cell death [22]. The mRNAs were most significantly enriched in steroid biosynthesis pathway components (Figure 3D). Because of their lympholytic activities, glucocorticoids (GCs) are included in many anti-leukemia therapeutic regimens [22]. To investigate the mechanisms by which DHA exerts anti-leukemia effects, we further examined the steroid biosynthesis pathway. Previous studies showed that estrogen, a type of steroid, represses differentiation of multipotent hematopoietic stem cells into lymphoid and myeloid cells [23, 24]. Figure 3E shows the potential DHA-related mRNA and lncRNA target sites within the steroid biosynthesis pathway map. Four key constant candidate mRNA targets, including FDFT1, SQLE, MSMO1, and DHCR7, were regulated by four lncRNAs. lncRNA Fendrr (ENSG00000268388) regulated all four key mRNA targets. Fendrr downregulation is associated with poor prognosis in gastric cancer due to its downregulating on fibronectin 1 expression [25]. lncRNA CRNDE (ENSG00000245694) regulated FDFT1 and DHCR7. All four of these mRNAs were upregulated after DHA treatment.

### DHA candidate targets in other drugs

**Figure 4:**
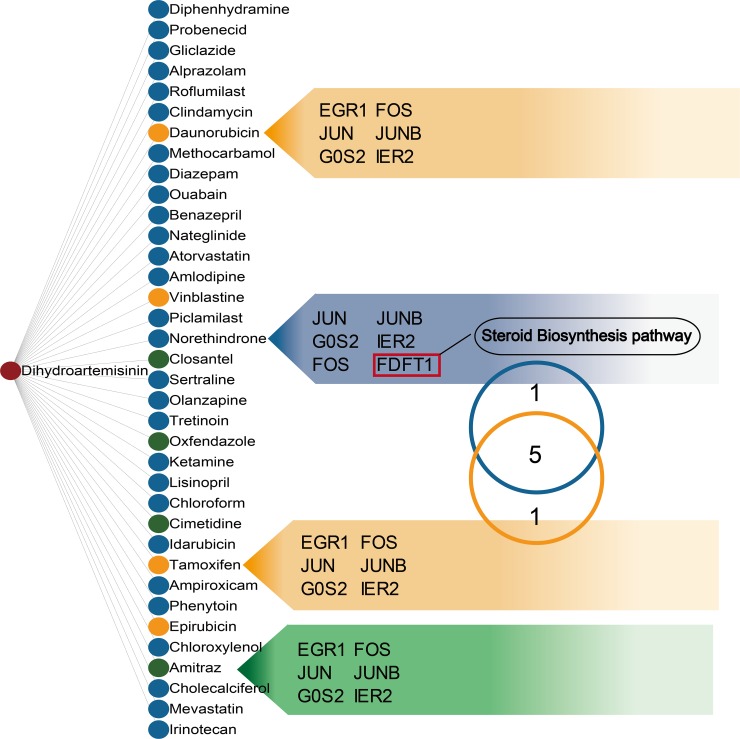
Drugs sharing common targets with DHA Two nodes are linked if they share common targets. Nodes in the list were ranked by drug similarity P-values. Green, yellow, and blue nodes represent anti-malarial, anti-cancer, and other drugs, respectively. Common target genes are listed. The Venn diagram shows that tamoxifen and norethindrone share common targets.

A hypergeometric test using the 34 dysregulated mRNAs revealed likely anti-CML DHA mechanisms by allowing us to study other drugs with similar targets. We assessed 36 drugs that were enriched in the same targets as DHA (Figure 4). These drugs produced distinct clinical outcomes and showed that DHA may have wide-ranging effects. As anticipated, the dysregulated mRNA targets in our study were enriched in some antiparasitic drugs, such as closantel, oxfendazole, and amitraz, which have anti-malarial activities similar to those of DHA. We also observed enrichment in some anti-cancer drugs, including daunorubicin, vinblastine, tamoxifen, and epirubicin, indicating that the candidate DHA targets were associated with cancer. Tamoxifen, used to prevent breast cancer by regulating estrogen, shared six target genes with DHA. Previous studies revealed that tamoxifen affects both primitive normal and malignant hematopoietic cells [26]. Our functional analysis also associated these DHA targets with steroid biosynthesis. Norethindrone, an estrogen regulated drug, shared six targets with DHA and five with tamoxifen. The common target gene, FDFT1, acts upstream of the steroid biosynthesis pathway. We inferred that DHA exerts anti-cancer effects in CML K562 cells by influencing steroid biosynthesis.

## DISCUSSION

Leukemia is characterized by abnormal growth of cells of the myeloid or lymphoid lineage. The pathogenesis of this disease is complex and likely includes chromosomal abnormalities, transcription factor alterations, and other factors [27]. Leukemias are categorized into four major types, including chronic lymphoblastic leukemia (CLL), chronic myeloid leukemia (CML), acute myeloid leukemia (AML), and acute lymphoblastic leukemia (ALL). CML is a hematopoietic stem cell-based disease, and 95% of cases arise as a result of the Philadelphia chromosome [28]. Effective treatment strategies and drugs differ between distinct leukemia types. Imatinib and interferon-α are primary treatment drugs for standard risk CML [29]. ALL is most commonly treated using GCs, such as dexamethasone and prednisolone [30]. However, improved treatment outcomes, especially for CML, have been hampered by drug resistance due to different biological and clinical features in patients, and enormous economic costs [31]. Novel and effective chemotherapeutic agents for the treatment of CML are urgently needed.

Artemisinin, known for its anti-malarial activity, reportedly has antitumor effects [32, 33]. In a test of the NC160 cell lines, leukemia cells were the most sensitive to artemisinins, and one of the DHA ester stereoisomers, ARTEST1, was the most active compound [34]. DHA was shown to induce apoptosis in AML cells [35, 36], but the effect of DHA on CML was not well understood. Our study used K562 cells derived from a patient in the acute transformation phase of CML to explore the DHA effect on CML. Ours is the first study to identify differences in lncRNA expression after DHA treatment in CML cells. Previous studies have shown essential roles for lncRNAs in human diseases [37, 38], and other groups have assessed disease pathogenesis through integrated mRNA-lncRNA double expression profiles [39]. Here, we constructed a DHA-associated lncRNA-mRNA interaction network to explore the mechanisms by which DHA exerts its anti-CML effects. We identified lncRNAs and mRNAs differentially expressed following DHA treatment in both pure and hemin-induced K562 cells. Some of these mRNAs and lncRNAs play key roles in steroid biosynthesis, indicating that DHA impacts CML cells by influencing this biological process. Previous studies showed that the steroid estrogen represses differentiation of multipotent hematopoietic stem cells into lymphoid and myeloid cells [23, 24]. Estrogen has also been associated with regulating the differentiation of pluripotent hematopoietic progenitor cells [40]. In future study, we will confirmed some key mRNAs and lncRNAs were differential expression by further experiment.

We then computationally examined drugs with targets similar to those of DHA to explore DHA activity in CML cells. Aside from other anti-malarial drugs, we found that the DHA candidate targets identified in our study were also enriched in some anti-cancer drugs. Tamoxifen and DHA shared six targets. Tamoxifen, an anti-estrogen drug, is used to treat breast and ovarian cancers [41], and reportedly regulates both primitive normal and malignant hematopoietic cells [26]. Norethindrone, a synthetic progestational hormone used in oral contraceptives and to treat endometriosis, also shared six targets with DHA. Five of these targets were also shared with Tamoxifen, and one, FDFT1, is a key player in the steroid biosynthesis process. We inferred that, similar to tamoxifen, DHA affects CML K562 cells by influencing estrogen levels.

In summary, we identified candidate DHA-targeted mRNAs and lncRNAs, and constructed a DHA-related mRNA-lncRNA regulation network. Candidate mRNAs were enriched in steroid biosynthesis pathway components and were regulated by several lncRNAs. DHA and tamoxifen shared six common targets, indicating that they may act against CML through similar mechanisms, including estrogen regulation. Our findings suggest that DHA may be effective not only in treating malaria, but also as an anti-CML chemotherapeutic.

## MATERIALS AND METHODS

### Materials

Dihydroartemisinin (DHA) was purchased from Saan Chemical Technology Co., Ltd. (Shanghai, China), dissolved in DMSO to make a 1000 mM stock solution, and stored at 4°C.

### Cell culture

The human leukemia cell line, K562, was obtained from the Baoruyi Biotechnology Co., Ltd. (Beijing, China), and was cultured in RPMI 1640 medium with 10% fetal calf serum and 1% penicillin-streptomycin solution. Cells were maintained at 37°C in humidified air with 5% CO_2_. All reagents were from Corning, USA, and cells were free of mycoplasma.

### Cell treatments

Subconfluent cells (60–70%) in complete cell culture medium were treated with 1 or 10 μM DHA in DMSO; control cells were treated with 0.1% DMSO. Subsequent experiments were repeated three times. Cells were induced using hemin (Jingkehongda Biological Technology Co., Ltd., Beijing, China). The hemin stock solution concentration was 5 mM in 0.1 M NaOH, and the final treatment concentration was 40 uM.

### RNA extraction

Total RNA was extracted and isolated using Trizol according to the manufacturer's instructions. RNA quantity and quality were measured using a NanoDrop spectrophotometer.

### lncRNA and mRNA sequencing

Libraries were sequenced on the Illumina HiSeq 2500 platform using the 125-bp pair-end sequencing strategy. Raw data (raw reads) in were processed in fastq format. Clean data (clean reads) were obtained by removing reads containing adapters or poly-N regions, and low quality reads from the raw data. Q20, Q30, and GC content values were calculated from clean data.

### Read mapping and expression analysis

Reference genome and gene model annotation files were downloaded directly from the University of California, Santa Cruz Genome Browser website (http://genome.ucsc.edu). The reference genome index was built using Bowtie v2.0.6 and paired-end clean reads were aligned to the reference genome using TopHat v2.0.9. Mapped reads from each sample were assembled using both Scripture (beta2) [42] and Cufflinks (v2.1.1) in a reference-based approach [43]. Previously unknown lncRNAs expressed in K562 cells were also identified. Transcripts >200 nucleotides (nt) were identified as lncRNAs if they did not overlap with known genomic annotations from the Ensembl Database, and no coding potential was identified via PhyloCSF [44, 45]. Cuffdiff (v2.1.1) was used to calculate FPKMs of both lncRNAs and coding genes in each sample. Gene fragments per kilobase of transcript per million mapped reads (FPKMs) were computed by summing the FPKMs of transcripts in each gene group. FPKM was calculated based on the length of the fragments and the reads count mapped to a given fragment.

### Identifying dysregulated mRNAs and lncRNAs

We used *t*-tests to identify lncRNAs and mRNAs dysregulated within four comparisons: (1) pure K562 cells vs. K562 cells treated with 1 uM DHA; (2) pure K562 cells vs. K562 cells treated with 10 uM DHA; (3) hemin-induced K562 cells vs. hemin-induced K562 cells treated with 1 uM DHA; and (4) hemin-induced K562 cells vs. hemin-induced K562 cells treated with 10 uM DHA. P<0.05 was considered a significant difference. We classified differentially expressed mRNAs and lncRNAs as upregulated or downregulated based on fold change values (fold change >2 and <0.5, respectively). The intersections of dysregulated mRNAs and lncRNAs in the four comparisons were identified as candidate targets of DHA.

### lncRNA-mRNA interaction network construction

The starBase, RAID v2.0, and NPInter databases were used to identify lncRNA-mRNA interaction networks [46–48]. 81,186 non-redundant, validated lncRNA-mRNA pairs were identified. Among these interactions, 428 lncRNA-mRNA pairs were associated with 34 and eight differentially expressed mRNAs and lncRNAs, respectively. Cytoscape software (version 3.2.1) was used to construct and illustrate the network [49].

### Functional enrichment analysis

Gene Ontology (GO) and KEGG pathways enrichment analyses were performed via the Enrichr tool online web server using default parameters [50]. We obtained significantly enriched GO terms (FDR<0.05) and KEGG pathways (P<0.1).

### Drug target genes enrichment analysis

A hypergeometric test was used to calculate the significance of enriched genes with respect to drugs. If the entire genome had a total of N genes, of which n were involved with the target genes under investigation, and a total of m DHA candidate genes, of which k were involved with the same drug target genes, then the P*-*value for the enrichment of that drug was calculated as follows:

$$P=1u\overset{k}{\underset{i=0}{\sum }}\frac{\left(\begin{array}{c} n\\ i \end{array}\right)\left(\begin{array}{c} N-n\\ m-i \end{array}\right)}{\left(\begin{array}{c} N\\ m \end{array}\right)}$$

Drugs with P<0.01 were considered significantly enriched.

## SUPPLEMENTARY MATERIALS FIGURES AND TABLES




