# Supplementary material for: Dihydroartemisinin-regulated mRNAs and lncRNAs in chronic myeloid leukemia
Source: Oncotarget. 2017 Dec 15;9(2):2543–52. doi: 10.18632/oncotarget.23274 (PMC5788658; doi:10.18632/oncotarget.23274)
Supplement: Supplementary file 1 [file oncotarget-09-2543-s001.pdf]

Dihydroartemisinin-regulated mRNAs and lncRNAs in chronic myeloid leukemia

SUPPLEMENTARY MATERIALS

Supplementary Table 1: Experimental confirmed leukemia-related mRNAs.

See Supplementary File 1
